# Supplementary material for: A Eucalyptus Pht1 Family Gene EgPT8 Is Essential for Arbuscule Elongation of Rhizophagus irregularis
Source: Microbiol Spectr. 2022 Oct 13;10(6):e01470-22. doi: 10.1128/spectrum.01470-22 (PMC9769952; doi:10.1128/spectrum.01470-22)
Supplement: Supplemental file 1 — Supplemental material. Download spectrum.01470-22-s0001.pdf, PDF file, 1.9 MB [file spectrum.01470-22-s0001.pdf]

Supplemental Material

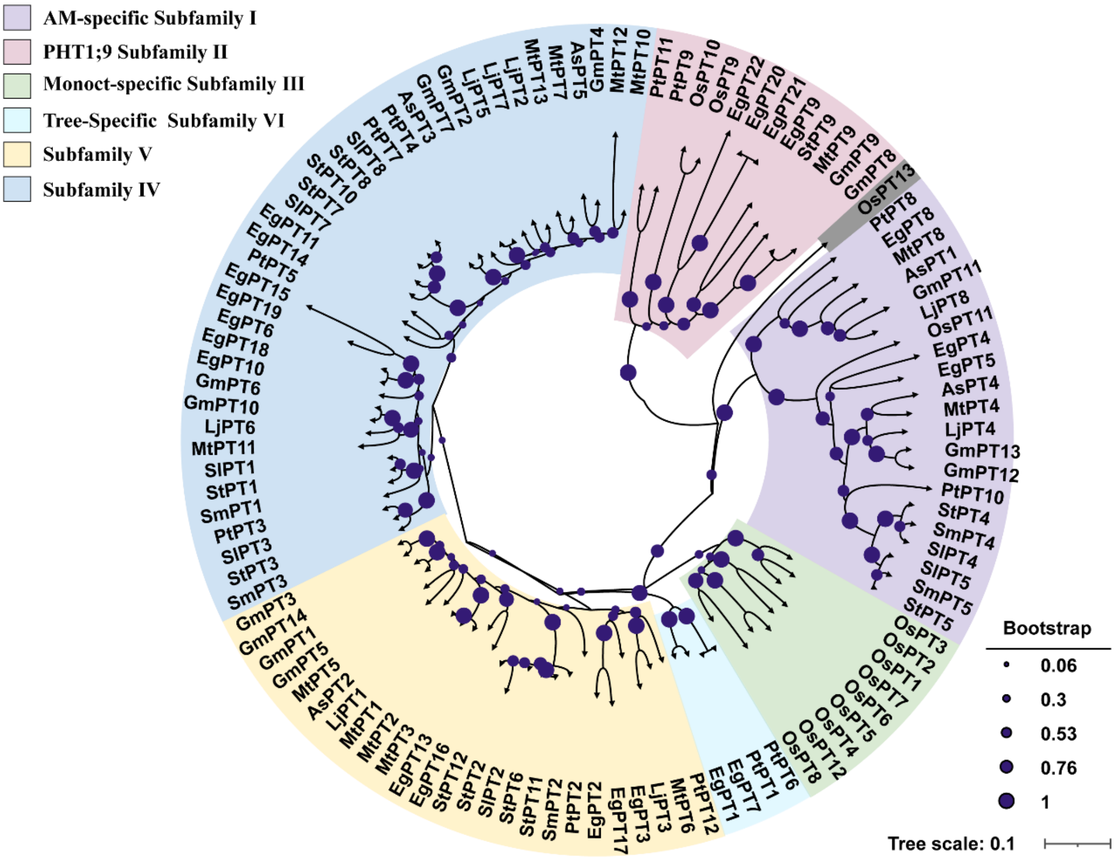

Fig. S1 Phylogenetic analysis of *E. grandis* Pht1 Pi transporters and other plant species Pht1 homologs.

An unrooted phylogenetic tree of Pht1 transporters was constructed using the neighbor-joining method with MEGA 7.0 program. Bootstrap values (range 0.06-1) are from 1000 replications. Corresponding plant species are: *E. grandis*, Soybean (*Glycine* max), tomato (*Solanaceae lycopersicum*), potato (*Solanaceae tuberosum*), *Populus trichocarpa*, Rice (*Oryza sativa*), eggplant (*Solanaceae melongena*), *M. truncatula*, *A. sinicus*, and *L. japonicus*. Accession numbers are given in Supplemental Materials Tab. S1.

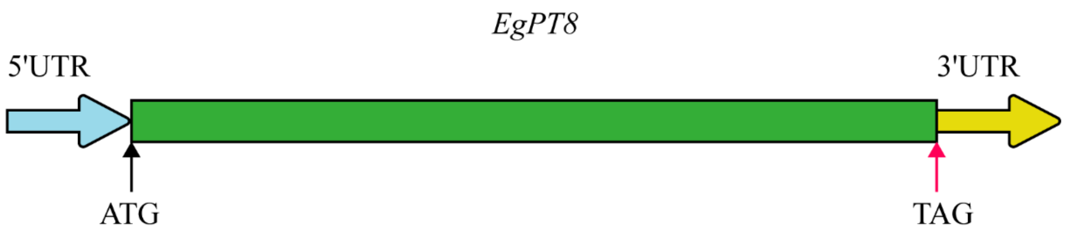

Fig. S2 Gene structure of *E. grandis EgPT8*.

The *EgPT8* gene corresponds to contains only one exon. The blue arrow and yellow arrow indicate untranslated regions (UTRs). The black arrow and red arrow are represented the translation initiation site and translation termination site, respectively.

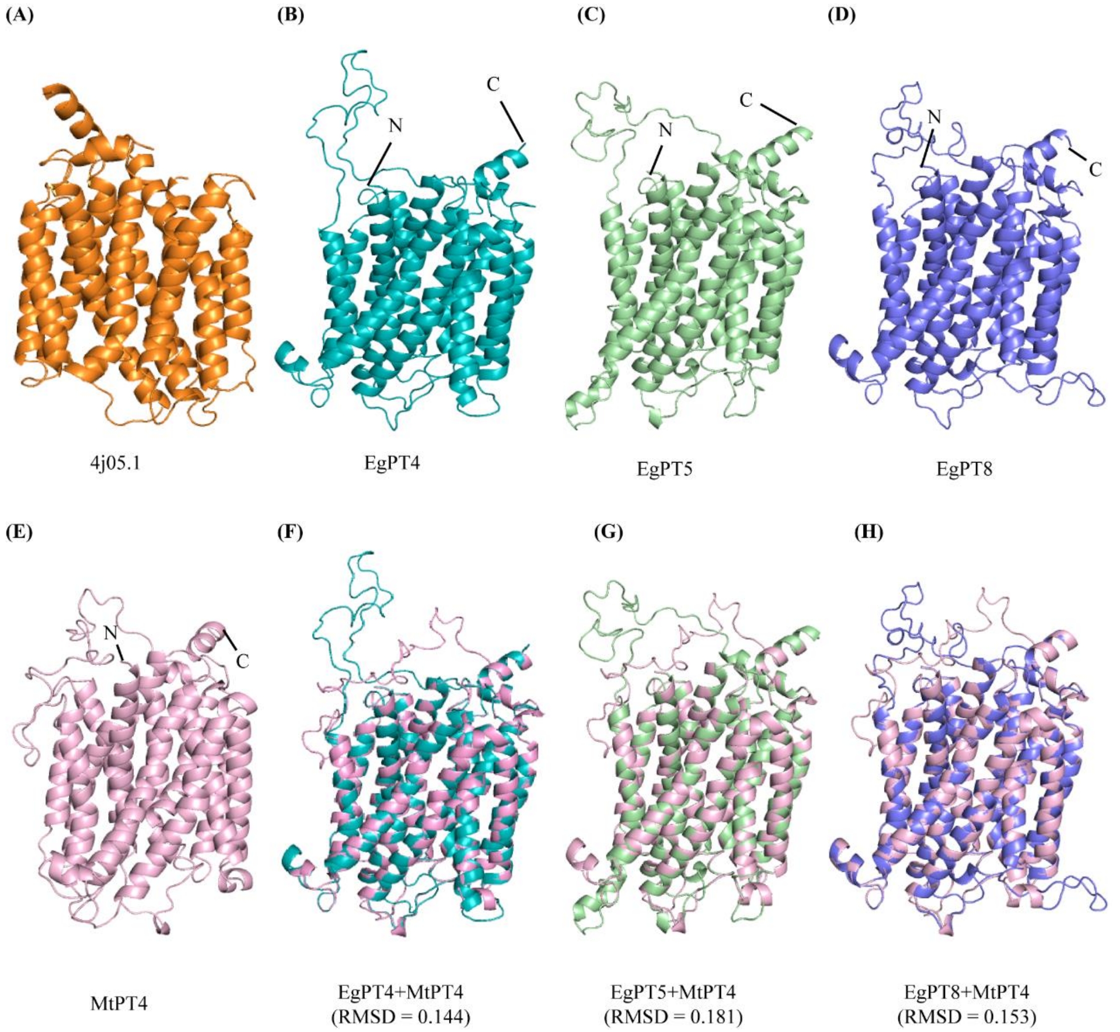

Fig. S3 Conservation of protein 3D structures of EgPT4, EgPT5, EgPT8 and MtPT4.

(**A**) The homology modeling template of EgPT4, EgPT5, EgPT8 and MtPT4, the corresponding PDB ID is 4J05.1. (**B-E**) The protein 3D structure of EgPT4, EgPT5, EgPT8, and MtPT4. (**G-H**) Overall structure comparison of three *E. grandis* genes and MtPT4. RMSD (Root Mean Square Deviation) value is displayed at the bottom.

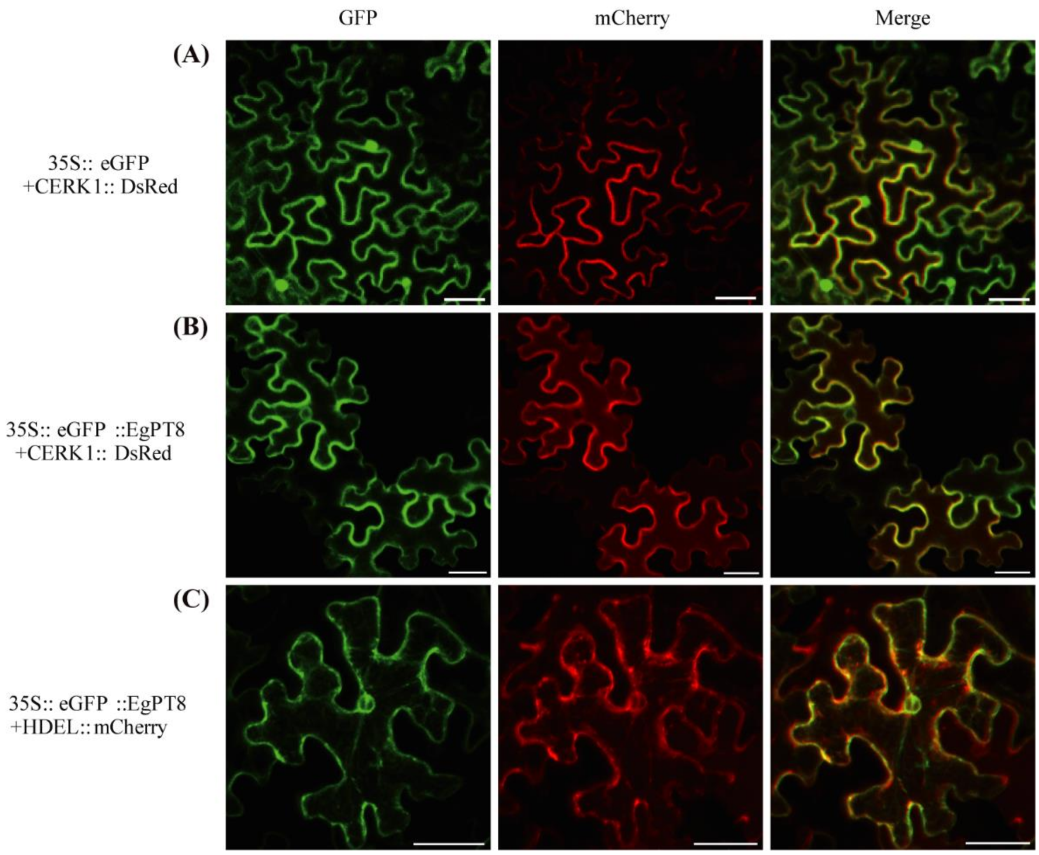

Fig. S4 Subcellular localization analysis of the EgPT5 and EgPT8 in tobacco.

The coding sequence of EgPT8 was fused in-frame with the 3' end of the GFP reporter gene under the control of the cauliflower mosaic virus 35S promoter (35S::eGFP::EgPT8). Confocal laser scanning microscopy images of *N. benthamiana* leaf epidermal cells transiently coexpressing either 35S::eGFP (**A**) or constructs with the plasma membrane marker CERK1::DsRed (**B**), or the endoplasmic reticulum marker HDEL-mCherry (**C**) driven by the 35S promoter. Left panels: GFP channel; center panels: mCherry channel; right panels: Merged. Scale bars, 50 μm.

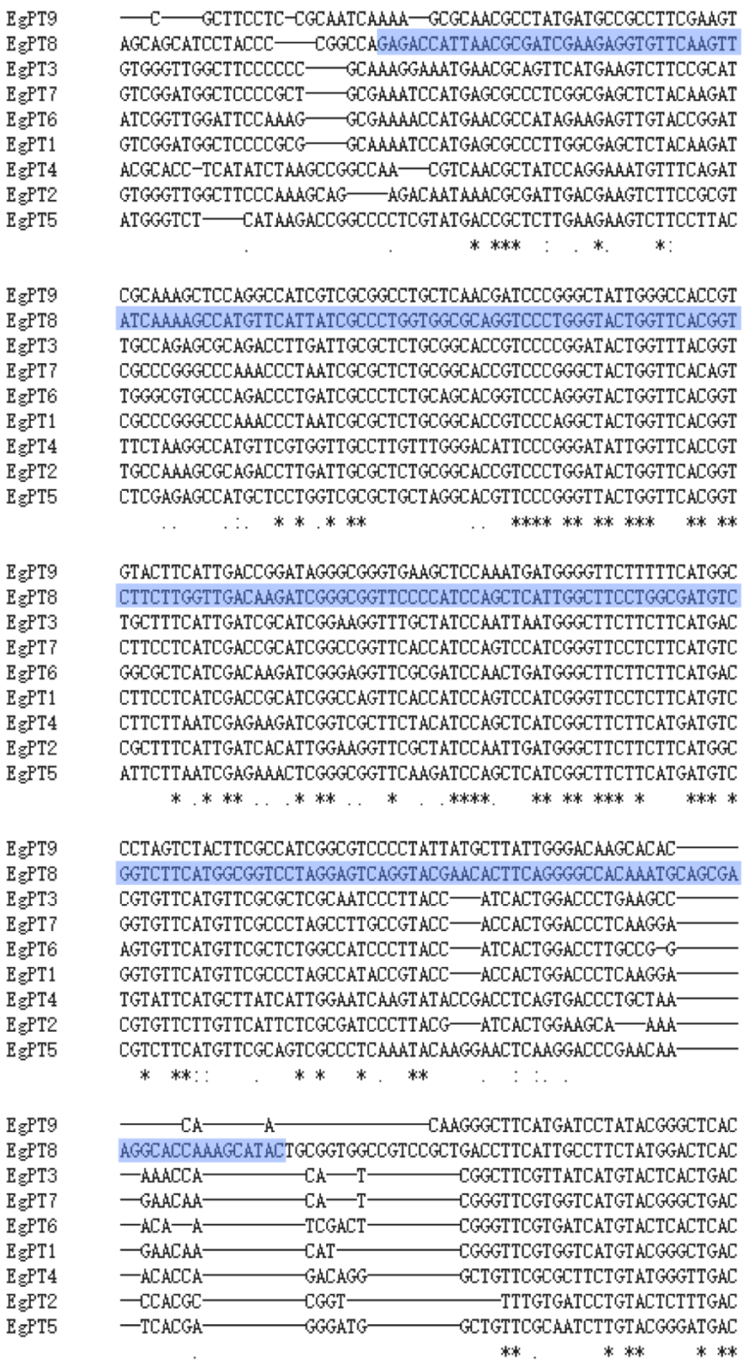

Fig. S5 The alignment of *EgPT8*-RNAi cDNA sequence with other *E. grandis* PHT1 family genes.

The RNAi sequence within the gene *EgPT8* is marked as blue shadow. Identical nucleotides are denoted with asterisks. Short lines represent blank arras. The corresponding sequence names are marked on the left.

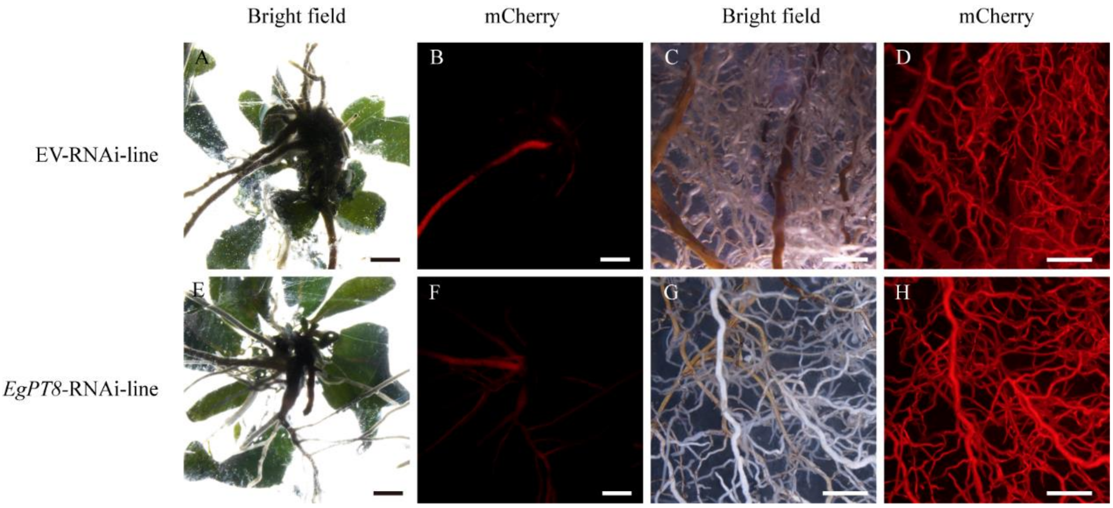

Fig. S6 Fluorescence microscopy images of the transgenic *E. grandis* roots.

(**A-B** and **E-F**) The composite *E. grandis* with strong hairy roots carrying the *RNAi-EgPT8* construct (**A**) or EV-RNAi empty vector (EV) (**E**) selected firstly from MS medium, based on the red fluorescence (**B** and **F**), respectively. (**C-D** and **G-H**) After 42 days of growth, the corresponding hairy roots were selected secondly in pot cultures on the basis of red fluorescence. Scale bars, 10 mm (**A**, **B**, **E**, **F**), 1 mm (**C**, **D**, **G**, **H**).

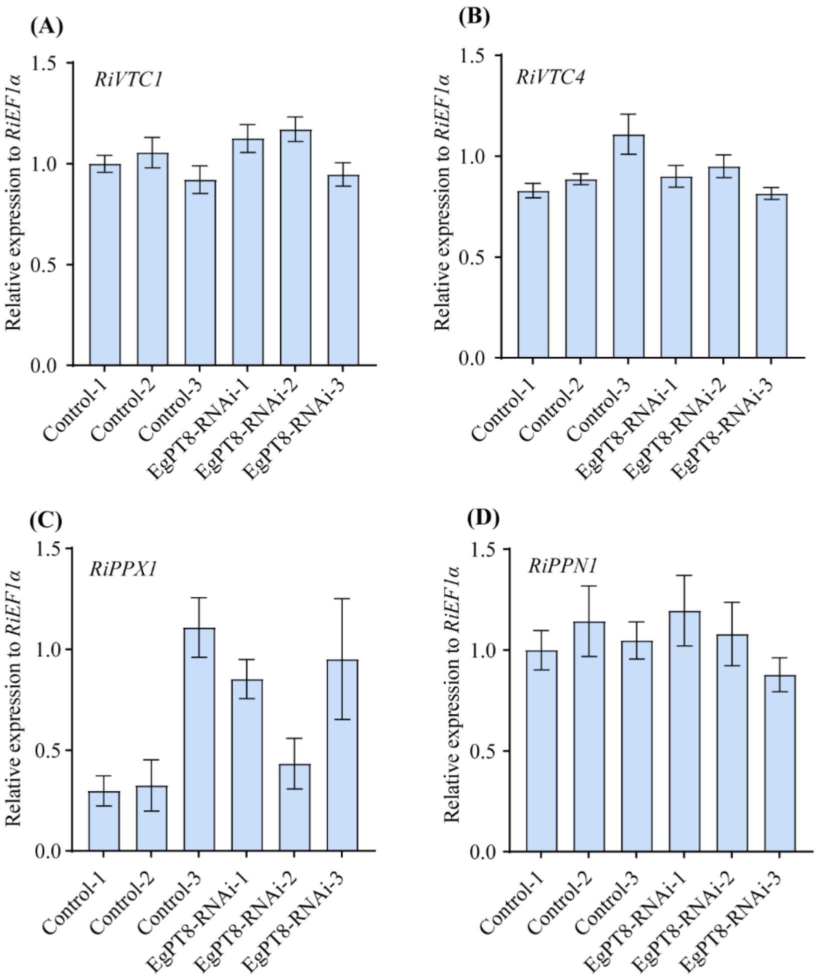

Fig. S7 Expression assay of the *R. irregularis* polyphosphate (polyP) synthetic and metabolism genes in the control and *EgPT8-RNAi* lines.

(**A**-**B**). Transcript of *RiVTC1*, *RiVTC4* involved in the vacuolar polyP accumulation in the control and *EgPT8-RNAi* mycorrhizal roots. (**C**-**D**). Quantitative RT-PCR analysis of *RiPPN1* and *RiPPX1* for polyP hydrolysis in the control and *EgPT8-RNAi* lines. Error bars indicate SD (n = 3). Control-1, Control-2 and Control-3 were set as the reference groups. Student's *t*-test.

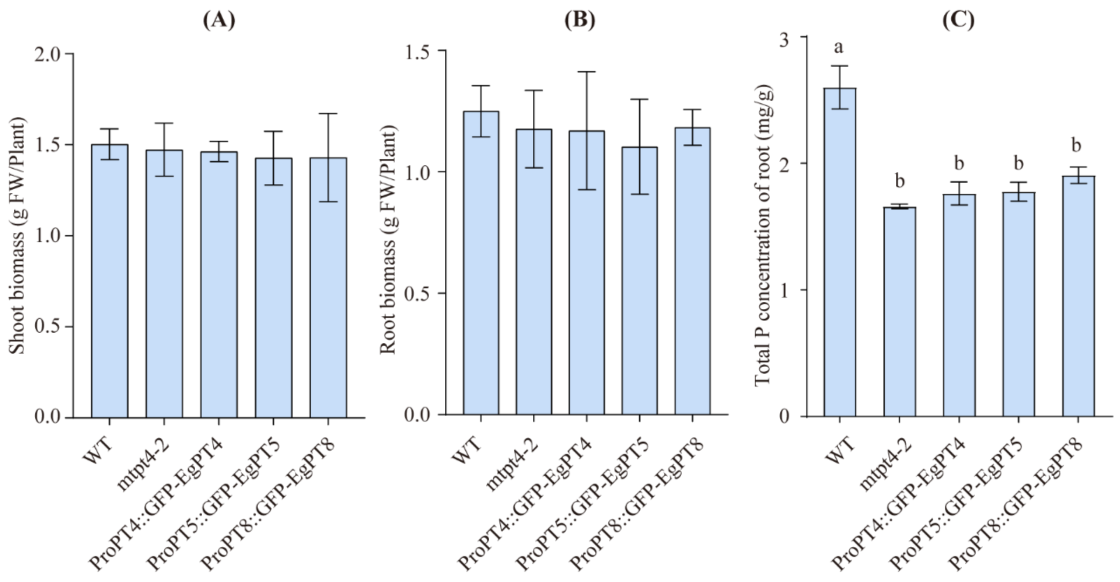

Fig. S8 Effects of complement of EgPT4, EgPT5, and EgPT8 function on plant growth and P uptake in the presence of AM fungal colonization.

(**A**-**B**) Shoot and root biomass were analyzed in WT, *mtpt4-2* mutant and transgenic lines. (**C**) Total P concentration of root was tested in in WT, *mtpt4-2* mutant and transgenic lines.

Table S1 Primers used in this study.

Primers used in this study

Primer Name	Prime sequences (from 5' to 3')	Use for
EgPT3-qF	TGAGGTTAGTGTGATTGCCAG	qRT-PRC
EgPT3-qR	AAGAGATCATACGCATCGGTG	qRT-PRC
EgPT4-qF	TGATCCTTTCCGCACTGTTC	qRT-PRC
EgPT4-qR	ATCCGCTTCTGGTTGTGTAG	qRT-PRC
EgPT5-qF	TTTCTGGGATAAGCACGGTC	qRT-PRC
EgPT5-qF	CTTTTGGGTCAGGTTTTGGC	qRT-PRC
EgPT8-qF	GAGTCAGGTACGAACACTTCAG	qRT-PRC
EgPT8-qR	CGGGACTATGAAGGTTGTGG	qRT-PRC
EgPT15-qF	CAAATCCTGCTCCGACCTATG	qRT-PRC
EgPT15-qR	CTGTTTCGGGCATTTTCATCC	qRT-PRC
EgUBI3-qF	TCACCTACGTCTACCAGAAGG	qRT-PRC
EgUBI3-qR	TCCTCGAAAGCTGTAAACATGG	qRT-PRC
RiVTC1-qF	GGAGAGCGACCAAGATAAGAAAG	qRT-PRC
RiVTC1-qR	AATTAGTTACCACGGCACCC	qRT-PRC
RiVTC2-qF	GATGAAGGAGAACAGGAGCC	qRT-PRC
RiVTC2-qR	ATGGAAGAGAAGGTGCATGG	qRT-PRC
RiVTC4-qF	TCCAACTACACCAGCCAATG	qRT-PRC
RiVTC4-qR	TGCATCTTCCTCATCTTCGTC	qRT-PRC
RiPPN1-qF	CGCGTAAACGTGGAATGAAG	qRT-PRC

RiPPN1-qR	AATGCCAATCGTCCGTATCT	qRT-PRC
RiPPX1-qF	AGACGAAAGATTATATCTCAACATCAAG	qRT-PRC
RiPPX1-qR	TGGCTAATCTAAAATCATCTCGGG	qRT-PRC
RiEF1α-qF	TGTTGCTTTCGTCCCAATATC	qRT-PRC
RiEF1α-qR	GGTTTATCGGTAGGTCGAG	qRT-PRC
PFL61-EgPT4-F	ATAAGAATGCGGCCGCATGGTTTCCAGCAGTTTAGCAG	Yeast Complementation
PFL61-EgPT4-R	ATAAGAATGCGGCCGCCTAAATGACAACTGACCGGCC	Yeast Complementation
PFL61-EgPT5-F	ATAAGAATGCGGCCGCATGGCGAACAATCTCGCAGTGC	Yeast Complementation
PFL61-EgPT5-R	ATAAGAATGCGGCCGCTTATTTGTAGTAGTGCCCCTCC	Yeast Complementation
PFL61-EgPT8-F	ATAAGAATGCGGCCGCATGGCAGATTCCAGCAGAGCCG	Yeast Complementation
PFL61-EgPT8-R	ATAAGAATGCGGCCGCCTAAACCATTTCCGTGTCCGG	Yeast Complementation
PUG36-EgPT4-F	CCGGAATTCATGGTTTCCAGCAGTTTAGCAG	Subcellular localization in yeast
PUG36-EgPT4-R	ACGCGTCGACCTAAATGACAACTGACCGGCC	Subcellular localization in yeast

PUG36-EgPT5-F	CCGGAATTCATGGCGAACAATCTCGCAGTGC	Subcellular localization in yeast
PUG36-EgPT5-R	ACGCGTCGACTTATTTGTAGTAGTGCCCCTCC	Subcellular localization in yeast
PUG36-EgPT8-F	CCGGAATTCATGGCAGATTCCAGCAGAGCCG	Subcellular localization in yeast
PUG36-EgPT8-R	ACGCGTCGACCTAAACCATTTCCGTGTCCGG	Subcellular localization in yeast
PUG36-ScPHO84-F	CCGGAATTCATGAGTTCCGTCAATAAAGAT	Subcellular localization in yeast
PUG36-ScPHO84-R	CGCGGATCCTGCTTCATGTTGAAGTTGAGA	Subcellular localization in yeast
pCANG-N-EgPT5-F	CGGGTTCGAAATCGATGGATCCATGGCGAACAATCTCGCAGTGC	Subcellular localization in tobacco
pCANG-N-EgPT5-R	CGCGTCCTAGGCTACGTAGGATCCTTATTTGTAGTAGTGCCCCTCC	Subcellular localization in tobacco
pCANG-N-EgPT8-F	CGGGTTCGAAATCGATGGATCCATGGCAGATTCCAGCAGAGCCG	Subcellular localization in tobacco
pCANG-N-EgPT8-R	CGCGTCCTAGGCTACGTAGGATCCCTAAACCATTTCCGTGTCCGG	Subcellular localization in tobacco
RANi-EgPT8-F	GGGGACAAGTTTGTACAAAAAAGCAGGCTGACCATTAACGCGATCGAAGAG	RNAi

RNAi-EgPT8-R	GGGGACCACTTTGTACAAGAAAGCTG GGTGTATGCTTTGGTGCCTTCGCTG	RNAi
1305-eGFP-ORF-F	GATCCTCTAGAGTCGACCTGCAGATG GTGAGCAAGGGCGAGGAG	Subcellular localization (pCAMBIA1305)
1305-eGFP-ORF-R	GGTGATTTTTGCGGACCTGCAGGAATT CCCGATCTAGTAACATAG	Subcellular localization (pCAMBIA1306)
pCambia1305-EgPT4-F	CAGCTATGACCATGATTACGAATTCTG CATAACTTGGTCCGTGATGGC	Subcellular localization (pCAMBIA1307)
pCambia1305-EgPT4-R	ACTCTAGAGGATCCCCGGGTACCTGA TAACTTCCTTCTCTCTCGGC	Subcellular localization (pCAMBIA1308)
pCambia1305-EgPT5-F	CAGCTATGACCATGATTACGAATTCG GAGTACAGGTCAATGGACCAAG	Subcellular localization (pCAMBIA1309)
pCambia1305-EgPT5-R	ACTCTAGAGGATCCCCGGGTACCTGA CGATGGCTTTTGCTCTGTTAG	Subcellular localization (pCAMBIA1310)
pCambia1305-EgPT8-F	CAGCTATGACCATGATTACGAATTCA AGAGAGCTAACCAGAAGTAGCC	Subcellular localization (pCAMBIA1311)
pCambia1305-EgPT8-R	ACTCTAGAGGATCCCCGGGTACCGGC TGAAATGCTCTACTCCCCCTAG	Subcellular localization (pCAMBIA1312)

Note: The underline indicates the sequence of the restriction site.

Table S2 Accession number of Pht1 sequences.

Species	Protein names	Accession Numbers
Eucalyptus grandis	EgPT1	ON012821
	EgPT2	ON012822
	EgPT3	ON012823
	EgPT4	ON012824
	EgPT5	ON012825
	EgPT6	ON012826
	EgPT7	ON012827
	EgPT8	ON012828
	EgPT9	ON012829
	EgPT10	ON012830
	EgPT11	ON012831
	EgPT13	ON012833
	EgPT14	ON012834
	EgPT15	ON012835
	EgPT16	ON012836
	EgPT17	ON012837
	EgPT18	ON012838
	EgPT19	ON012839

	EgPT20	ON012840
	EgPT21	ON012841
	EgPT22	ON012842
	SlPT1	NP_001234361.2
	SlPT2	NP_001234043.1
	SlPT3	NP_001318089.1
Solanum lycopersicum	SlPT4	NP_001234674.2
	SlPT5	XP_004240951.1
	SlPT7	XP_004247235.1
	SlPT8	XP_004240760.1
	MtPT1	XP_003590039.1
	MtPT2	XP_003590043.1
	MtPT3	XP_003590037.1
	MtPT4	XP_013466381.1
	MtPT5	XP_003590758.1
Medicago truncatula	MtPT6	XP_003601529.1
	MtPT7	XP_013449893.1
	MtPT8	XP_003615445.1
	MtPT9	XP_003607875.1
	MtPT10	XP_013468493.1
	MtPT11	XP_003590759.2

	MtPT12	XP_013468492.1
	MtPT13	XP_013449892.1
Populus trichocarpa	PtPT1	XP_006383468.2
	PtPT2	XP_006378342.1
	PtPT3	XP_024465249.1
	PtPT4	XP_002306844.3
	PtPT5	XP_002302047.1
	PtPT6	XP_002306623.2
	PtPT7	XP_002306845.2
	PtPT8	XP_024446611.1
	PtPT9	XP_002300626.3
	PtPT10	XP_006374329.2
	PtPT11	XP_002307816.2
	PtPT12	XP_002300153.3
Lotus japonicus	LjPT1	Lj2g3v1455410.1
	LjPT2	Lj1g3v4483780.1
	LjPT3	Lj6g3v0030000.1
	LjPT4	Lj1g3v0948470.1
	LjPT5	Lj0g3v0003149.1
	LjPT6	Lj5g3v1171160.1
	LjPT7	Lj0g3v0162149.1

	LjPT8	Lj2g3v2172990.1
Oryza sativa	OsPT1	AAN39042.1
	OsPT2	AAN39043.1
	OsPT3	AAN39044.1
	OsPT4	AAN39045.1
	OsPT5	AAN39046.1
	OsPT6	AAN39047.1
	OsPT7	AAN39048.1
	OsPT8	AAN39049.1
	OsPT9	AAN39050.1
	OsPT10	AAN39051.1
	OsPT11	AAN39052.1
	OsPT12	AAN39053.1
	OsPT13	AAN39054.1
Glycine max	GmPT1	NP_001240032.1
	GmPT2	NP_001304639.2
	GmPT3	NP_001341396.1
	GmPT4	NP_001304588.2
	GmPT5	NP_001240239.1
	GmPT6	NP_001239971.1
	GmPT7	NP_001254802.1

	GmPT8	NP_001345390.1
	GmPT9	NP_001241574.1
	GmPT10	NP_001240957.1
	GmPT11	NP_001239765.1
	GmPT12	NP_001241127.1
	GmPT13	NP_001241400.1
	GmPT14	NP_001241164.1
Solanum tuberosum	StPT1	NP_001275200.1
	StPT2	CAA67396.1
	StPT3	XP_006354489.1
	StPT4	XP_006360181.1
	StPT5	XP_006360180.1
	StPT6	XP_006356064.1
	StPT7	XP_006351654.1
	StPT8	XP_015166067.1
	StPT9	XP_006342170.1
	StPT10	XP_006358452.1
	StPT11	XP_006356063.1
	StPT12	XP_006356062.1
Solanum melongena	SmPT1	ABK63959.1
	SmPT2	ABK63961.1

	SmPT3	ABK63963.1
	SmPT4	ABK63966.1
	SmPT5	ABK63969.1
	AsPT1	AFU50500.1
	AsPT2	AFU50501.1
Astragalus sinicus	AsPT3	AFU50503.1
	AsPT4	AFU50504.1
	AsPT5	AFU50505.1
